# Perceived Cognitive Function and Mood in Spanish Language Hispanic and Latino Family Caregivers

**DOI:** 10.1093/geroni/igaf122.3636

**Published:** 2025-12-31

**Authors:** Sophia Salinas, Liliana Ramirez Gomez, Paulina Gutierrez-Ramirez, Maria Galvez, Felipe Jain

**Affiliations:** Massachusetts General Hospital, Boston, Massachusetts, United States; Massachusetts General Hospital, Boston, Massachusetts, United States; Massachusetts General Hospital, Boston, Massachusetts, United States; Massachusetts General Hospital, Boston, Massachusetts, United States; Massachusetts General Hospital / Harvard Medical School, Boston, Massachusetts, United States

## Abstract

Spanish-speaking Hispanic and Latino family caregivers of individuals with dementia experience high levels of stress, depression, and emotional strain. These challenges can also impair cognitive functioning. Limited access to culturally and linguistically appropriate support compounds difficulties in Latino communities. We examined correlates of perceived cognitive function in Spanish language caregivers and how improvements in psychological symptoms after a mindfulness intervention correlated with improvements in perceived cognitive function. Data were combined from two pilot, open-label feasibility studies of mentalizing imagery therapy (N = 17), a mindfulness and guided imagery program delivered via telehealth. At baseline and follow-up, participants completed measures of depression, stress, caregiver burden, mindfulness, social support, well-being, and neurological quality of life (Neuro-QoL). Pearson correlation coefficients were calculated for baseline data and changes over time. At baseline, lower depressive symptoms, stress, and caregiver burden were significantly associated with higher Neuro-QoL scores (depression: r=–.447, p=.022; stress: r=-.396, p=.041; caregiver burden: r=–.459, p = 0.016). Improvements in well-being from before to after the trial were linked to better Neuro-QoL outcomes (r=.440, p=.025), but changes in mindfulness or social support were not associated with Neuro-QoL outcomes. These findings suggest that improving mental well-being is closely linked to improved perceptions of cognitive function in Spanish-speaking caregivers. However, targeting trait mindfulness as a means to improving perceived cognitive function in this population was not supported by this data. Culturally tailored interventions addressing multiple aspects of caregiver distress can enhance emotional well-being and cognitive health, offering a promising approach for underserved Spanish-language populations.

